# A Compact Broadband Common-Aperture Dual-Polarized Antenna for Drone Applications

**DOI:** 10.3390/mi16010048

**Published:** 2024-12-31

**Authors:** Xue-Ping Li, Chao-Liang He, Jun-Fei Ji, Meng-Bing Yang, Yan Zhang, An-Xue Zhang, Wei Li

**Affiliations:** 1 College of Electronic and Electrical Engineering, Henan Normal University, Xinxiang 453600, China; lixueping@htu.edu.cn (X.-P.L.); 15893820057@163.com (C.-L.H.); jfji2021@126.com (J.-F.J.); 15886778141@163.com (M.-B.Y.); 2022076@htu.edu.cn (Y.Z.); 2 Henan Key Laboratory of Optoelectronic Sensing Integrated Application, Henan Normal University, Xinxiang 453600, China; 3 School of Information and Communications Engineering, Xi’an Jiaotong University, Xi’an 710049, China; anxuezhang@mail.xjtu.edu.cn

**Keywords:** dual polarized, common aperture, wideband, axial ratio (AR) bandwidth

## Abstract

A novel common-aperture miniaturized antenna with wideband and dual-polarized characteristics is proposed, which consists of a circularly polarized (CP) and a linearly polarized (LP) antenna. The circularly polarized antenna stacked on the upper layer adopts asymmetrical ground and introduces the patch and T-type feed network. On this basis, the meshed reflector structure, which also works as a ground plane for the LP antenna, is added to reduce the influence on circular polarization and achieve directional radiation. The LP antenna stacked in the lower layer uses a monopole structure, and the coaxial feed line perforates the reflector, and thereby the common-aperture antennas are tightly stacked together from top to bottom. Simulation and test are in good accordance, and the results show that the two ports of the antenna are well matched in the range of 5.5 GHz to 7.8 GHz, where peak gains of 8.5 dB and 6 dB are realized for circular polarization and linear polarization, respectively. Moreover, the 3 dB axial ratio (AR) bandwidth of the CP antenna is 34.3% and the isolation between the two ports is better than 15 dB, suggesting potential applications in the relay platform or drone detection for signal transmission and reception.

## 1. Introduction

With the rapid development of wireless communication technology, unmanned aerial vehicles (UAVs) with superior channel capacity and high reliability has gradually been put forward. The communication requirements of these devices during normal operation need more compact and high-performance antenna. To meet the practical need of such applications, broadband antennas with dual-polarization are taken as a proper option.

As is known, different types of dual-polarization antennas have been proposed and verified in recent publications, and since the polarization type of antennas is determined by their working modes, many dual-polarization antennas have the same feed structure or radiation structure to achieve dual-linear polarization or double-circular polarization [1,2,3,4]. For example, two shaped strips serve as a feed-in structure, and the symmetrical radiation mechanism allows the antenna to operate in the same frequency band, achieving similar directional radiation patterns and two-line polarization [1]. The air through holes inside the dielectric resonator antenna (DRA) is cleverly arranged by taking advantage of the diversity of dielectrics in [2]. The same feed structure is used to achieve two polarization modes, and works in similar operating frequency bands (6.67–6.78/4.83–6.99). Furthermore, the omnidirectional dual polarization in [3] consists of a horizontally polarized cavity rear slot and a vertically polarized folded slot, which can achieve dual-polarized omnidirectional radiation (2.34–2.72/2.39–2.49 GHz). In the latter, dual polarization is attained by introducing the orthogonal linear polarization with the same radiant structure in the same frequency band, but these designs are relatively large, which is not conducive to the integrated design. In addition, most of the previously reported dual-polarized broadband antennas are suspended cross-dipole antennas or floating patch antennas [5,6,7,8]. All of these antennas typically have the characteristics of more complex feeding schemes, such as differential feeds or the need for a balancer and phase shifter for dual-polarization operation.

To reduce antenna size and improve performance, stacked antennas are widely used. The advantages of more flexible combination of stacked dual-polarization antennas have been established, and different polarization modes can be achieved by superimposing multilayer structures [9,10,11]. In [9], complex networks and multiple patches are stacked on different dielectric plates to excite different linear polarization modes. By using multi-dipole stacking, as in [10], different dipoles can be operated in different frequency bands. There are also antennas that use multilayer dielectrics stacked with different slots [11], which are combined with the feed network to achieve different polarization modes in the same frequency band. This stacking structure is a new approach in the development of dual-polarization antennas, and can greatly reduce the strong coupling problem caused by the same radiation mode through using different excitation or radiation structures. Based on the advantages of this structure, linear/circularly polarized antennas have been proposed. In [12], a linear polarization patch and circular polarization patch are stacked to achieve double-band and dual-polarization characteristics, and 10 dB impedance bandwidth and high-band impedance bandwidth are 2.8% and 9.0%, respectively. The implementation of broadband circular polarization generally requires feeder networks, which also need to be added to the multilayer structure [13,14,15,16]. For example, an aperture-shared patch antenna [15] consists of an S-band circularly polarized (CP) antenna and an X-band linearly polarized (LP) antenna with axial ratio (AR) bandwidth up to 19.2%. The S-band uses a stacked structure to introduce the parasitic patch and feed network, and the parasitic patch is also used as the ground layer of the X-band. In the dual-polarization antenna studied in [16], to increase the 3 dB AR bandwidth, a complex feed network is used in a multilayer structure, and a wideband dual-polarization antenna is obtained by combining truncated patch and sequential rotation techniques, but the AR bandwidth is only 10.3%.

In this paper, a novel broadband common-aperture dual-polarized antenna with a compact design is proposed. To realize such an antenna, a CP antenna is stacked on a LP antenna, where the LP antenna adopts a monopole structure, and the CP antenna uses a patch and an improved T-type feed network. In addition, the ground plane of the monopole is also cleverly laid out with the grid that acts as a reflector for the CP antenna. Thus, the polarization bandwidth, impedance bandwidth, and size of CP antenna are improved. The designed antenna is easy to fabricate and can be applied in the relay platform or drone detection for signal transmission and reception. To further examine the antenna, one antenna prototype is manufactured and tested, and some deep analyses are conducted for better understanding.

## 2. Antenna Design

### 2.1. Configuration

**Figure 1:**
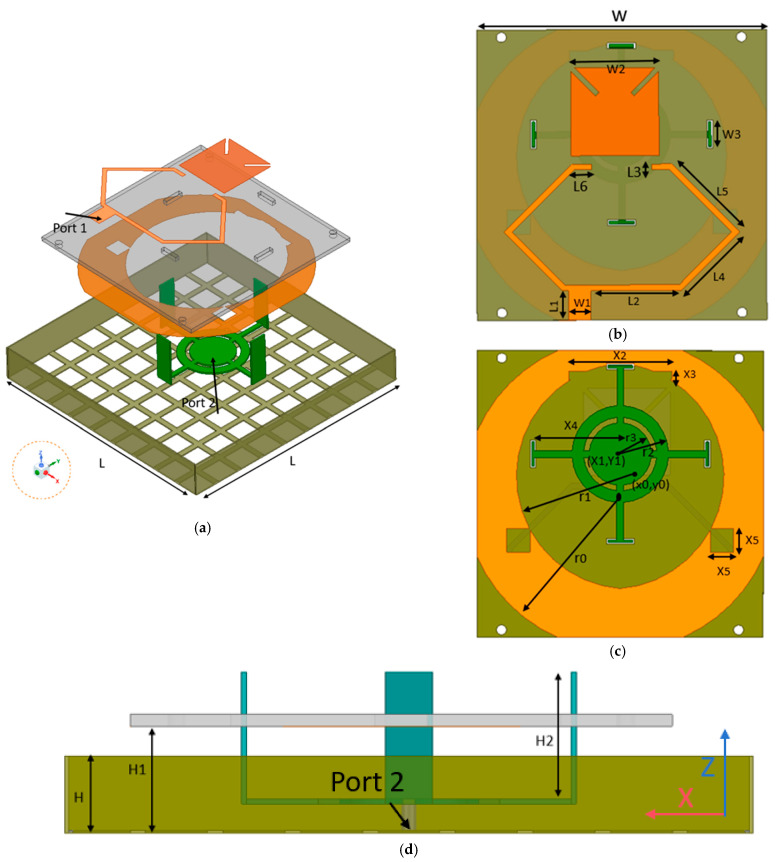
Geometry and dimensions of the proposed antenna. (**a**) Explosion view; (**b**) substrate top; (**c**) substrate back; (**d**) side view.

Figure 1 shows the configuration of the proposed antenna, which is composed of a substrate and metal layer, forming a multilayer structure. In addition, it has dual input ports, which can feed the signal to different radiation structures of linear and circular polarization.

As shown in Figure 1a, the LP antenna is a monopole that includes concentric circles and rings with radii *r*2 and *r*3 and curved branches in four directions. To adjust the impedance matching, the end of the branch with a width of *W*3 is bent upwards by 90° to form a folded monopole, which is fed coaxially, that is, the outer conductor is connected to the meshed metal cavity as ground plane, and the inner conductor is fed to the bottom circle center of the radiation structure. The CP antenna radiator is arranged above the weakly radiating part of the center for the monopole, and the dielectric plate is placed perpendicularly to the monopole with a dielectric constant of 2.2 and thickness of 0.762 mm, as shown in Figure 1b. In addition, the radiation patch and ground are printed on both sides of the square substrate, and one feed network is also located on the same side of the square patch. Moreover, there is an asymmetric T junction below the feeder network, and the length difference between the two sides is about λ/4. The microstrip lines on both sides are connected to the lower T junction at 135°, providing smooth input impedance and broad bandwidth. The curved microstrip lines connected on both sides are symmetrical with respect to the Y axis and the bent angle is 90°. The feeder network is placed in parallel with short branches at the two corners of the square, coupled with the patch. The patch is square and diagonally etched with a narrow gap to regulate the coupling current.

Figure 1c shows that the ground plane is annular-shaped as a whole, and the backplane is formed symmetrically around the Y axis after cutting by a ring with radii *r*0 and *r*1. Several additional slots are also added on the backplane to change the current distribution and increase the AR bandwidth. The CP radiating structure is perpendicular to the LP radiation structure, which reduces the spatial overlap and reduces the coupling. Thus, the antenna consists of two stacked antennas with different polarization modes. Broadband vertical polarization can be generated by a coaxial feed from port 2, and the CP antenna, which is perpendicular to the LP antenna with reflective metal cavities stacked at the bottom, is circularly polarized by a microstrip network feed from port 1. Table 1 demonstrate the concrete parameters of the proposed common-aperture dual-polarized antenna.

### 2.2. Working Principle

To better investigate the working mechanism of the dual-polarized antenna, the implementation of different polarization modes is discussed in the following. First of all, the antenna operates as a LP monopole at port 2, and its omnidirectional radiation in the 45° direction mainly comes from the uniformly distributed metal branches and the reflective cavity at the bottom. The folded metal branches work together with the reflective cavity at the bottom to form a uniform radiation pattern. Folding metal branches occupy less space while maintaining their radiation properties. In addition, connecting the folded structure with a concentric ring can improve the radiation mode of the antenna, enhancing the radiation effect and impedance matching performance of the antenna through its symmetry. The current flows vertically on the metal arm of the monopole, generating a vertical electric field component. Moreover, one end of the monopole antenna is connected to the metal grid ground plane, which affects the radiation of the antenna and further promotes the direction of the electric field, forming vertical polarization.

**Figure 2:**
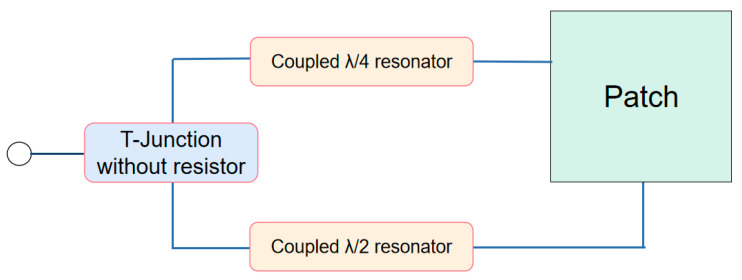
Topology of the proposed antenna.

The CP antenna is divided into two parts of equal amplitude and different phases by the feed network through power distribution. The feeder network consists of an equal non-inverting Wilkinson power divider and λ/4 resonator. Due to the difference in length of the two microstrip lines, the phase difference near the center frequency remains stable at −90°, exhibiting small ripples. The power divider is connected to the resonator by capacitive coupling. As shown in Figure 2, the feed network can make the incident wave convert to equal amplitude and different phases through the power allocation, and the patch can be excited through tight coupling to provide CP waves [17,18]. The end of the feed network with a certain symmetrical structure can be regarded as two monopoles that provide LP radiation. In order to obtain good circular polarization characteristics at 5.5 GHz, a T-shaped structure that produces a quarter-wavelength difference is designed, which connects to the microstrip line and increases the microstrip line spacing. The angle between the 90° curved microstrip line on both sides and the bottom T-shaped structure is 135°, which further widens the distance and weakens the coupling of the two microstrip lines on the basis of space saving. Moreover, two symmetrical ports of the feed network are parallel on the same side, each feed point is located in a square corner, and the coupling feeds of the patch are placed relative to each other. In order to make the CP characteristics more stable, the patch is shifted 0.8 mm to the left and the coupling length is finely adjusted to change the intensity of the current. After optimizing the length difference, a simple feed structure that can generate CP characteristics at the frequency point of 5.5 GHz is obtained.

**Figure 3:**
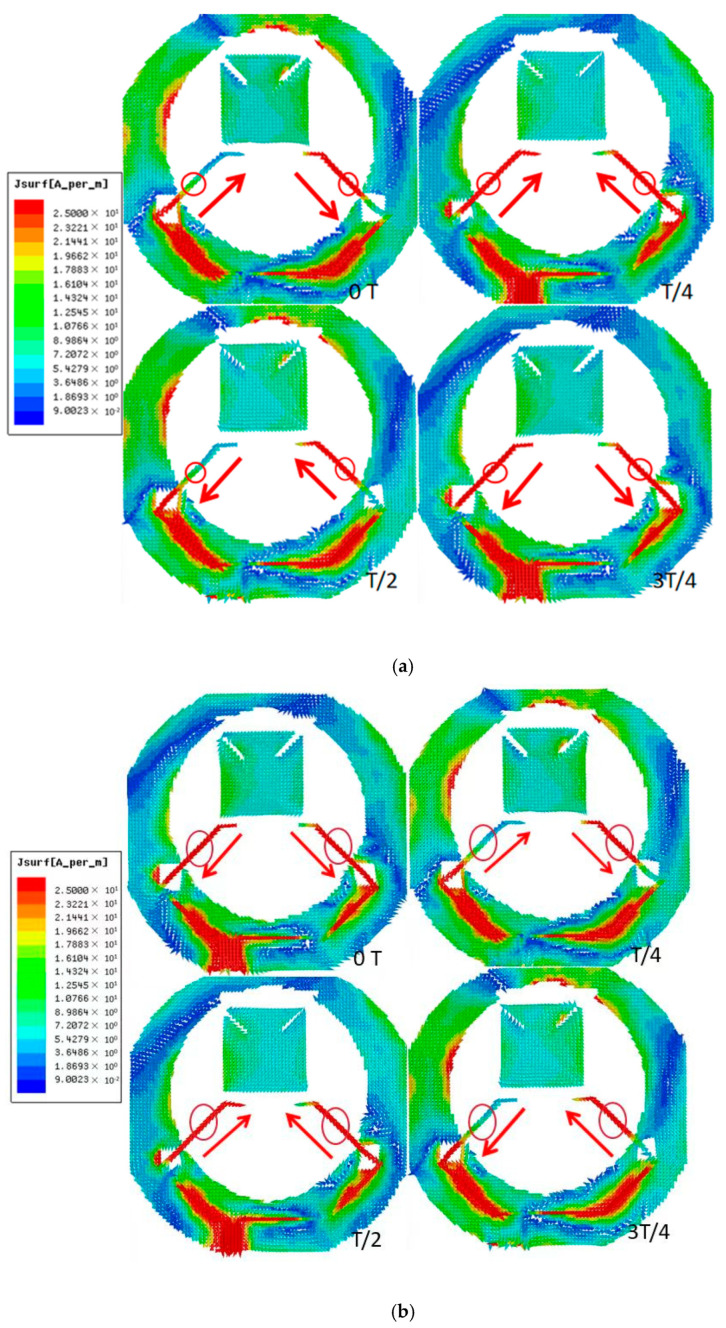
Surface current distribution from the front (+z). (**a**) 5.5 GHz; (**b**) 6.5 GHz.

To improve the overall CP characteristics, two narrow grooves with a length of 4.5 mm are etched diagonally on the square patch. Moreover, a slim groove at the top is introduced to change the current distribution on the floor. As shown in Figure 3, the current on the floor is mainly distributed at the bottom and inner loop of the ring. The current of port 1 is concentrated on both sides of the substrate. To further investigate the CP resonant modes, the surface current distributions of 5.5 and 6.5 GHz on the path are also displayed. The two orthogonal fundamental modes of TM01 and TM10 are excited simultaneously for different angular times. Therefore, right-hand circular polarization (RHCP) is obtained. At 5.5 GHz, when the antenna is working for 0°, the surface current distribution has some differences from the conventional TM01 because of the influence from the dual-coupled line, but the dominant surface current is mainly along the +*x*-axis, which is the same as TM01. When for 90°, TM10 is generated, which is towards +*y*-axis. In the same way, it can be concluded that the electric field of the antenna follows the rule in a cycle, and finally the RHCP radiation is formed.

**Figure 4:**
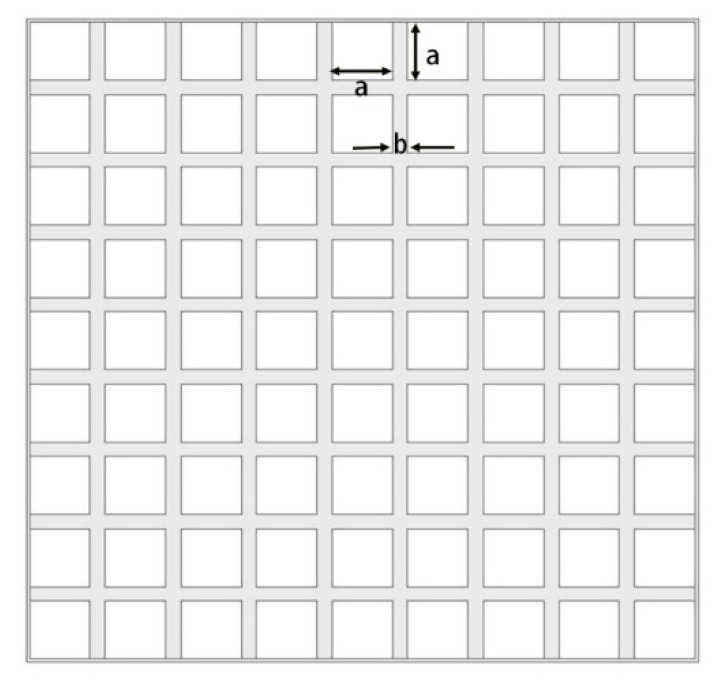
Structure of the proposed reflector.

Moreover, the use of a reflector to convert the bidirectional to unidirectional radiation can improve the overall gain of the antenna, and also produce good directional characteristics. Conventional reflectors are placed at least λ/4 away from the emitter to provide the phase delay of 180° for the reflected waves, and the height of the λ/4 reflector is too high to maintain the compactness of the stacked structure. To improve the omnidirectional radiation of port 2 and ensure that the circular polarization of port 1 is not affected, a special structural reflector is constructed. As shown in Figure 4, the meshed cell is designed to form a reactive impedance surface as a port reflector, and some extra inductance is introduced by the meshed cavity reflector. The array of metal strips is inductive in parallel with the *E* field, where the grid can be regarded as an LC resonant groove. Thus, the values of *L* and *C* can be roughly calculated as follows [19]:

(1) $$L=\mu_{0}\frac{a+b}{2\pi }\log(\csc\frac{\pi b}{2\left(a+b\right)})$$

(2) $$C=\varepsilon_{0}\frac{2\left(a+b\right)}{\pi }\log(\csc\frac{\pi a}{2\left(a+b\right)})$$

where *a* is the spatial width of the grid and *b* is the width of the grid strips. The calculated values of *a* and *b* are 4 mm and 1 mm, respectively. After using the grid metal mesh, the phase of the reflected wave is changed, and the distance between the reflected wave and the substrate can be reduced, even if the phase between the radiator and the reflector is less than 180°, but the total phase is close to 0° after superposition with the phase less than 180° generated by the distance. The cavity reflector makes the maximum radiation direction of the pattern for the LP antenna in the direction of θ = 45°, and the gain is 6 dB.

**Figure 5:**
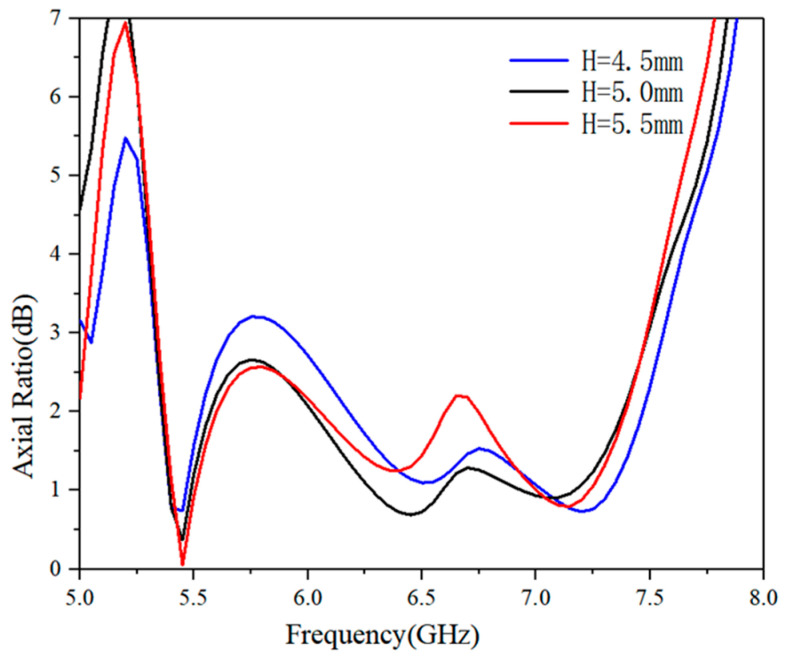
Simulated AR with different distances between the reflector and antenna.

Due to the CP capability affected by the reflector cavity height, we investigate the influence of the cavity height on the AR performance in Figure 5. When other parameters are kept constant, the optimized distance *H* is set from 4.5 mm to 5.5 mm, and the step size is 0.5 mm. As a whole, the corresponding AR can exhibit better performance with the increase in height. In the frequency band of 5.3–6.0 GHz, the optimal value is achieved at a height of 5.5 mm, but at around 6.75 GHz, the AR value is not ideal. When the height is 4.5 mm, a relatively poor AR can be observed in the low-frequency range, and the maximum value is 3.2 dB. Compared with the high frequency, the variation of the reflective cavity has a more significant impact on the AR at low frequency. Considering the AR performance and miniaturization of the antenna, the final value of *H* is set to 5 mm.

## 3. Results and Discussion

**Figure 6:**
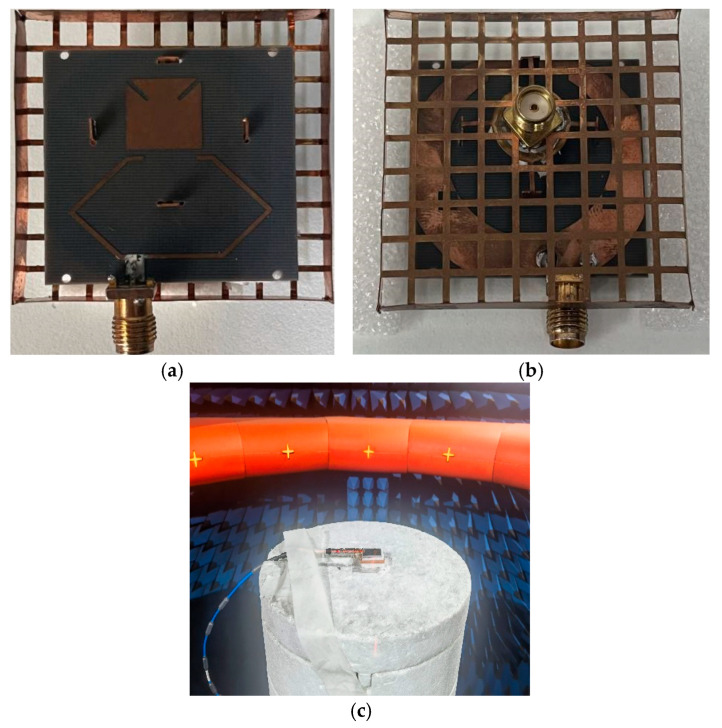
Photograph of the proposed antenna. (**a**) Top view; (**b**) back view; (**c**) test setup in the anechoic chamber.

In order to verify the designed antenna, the final prototype is manufactured using standard printed circuit board (PCB) technology, and relevant optimized values of parameters are exhibited in Table 1. Moreover, 50 Ω coaxial cables and microstrip lines are used to excite the proposed dual-polarized antenna. Finally, the antenna is measured in an anechoic chamber, as shown in Figure 6.

**Figure 7:**
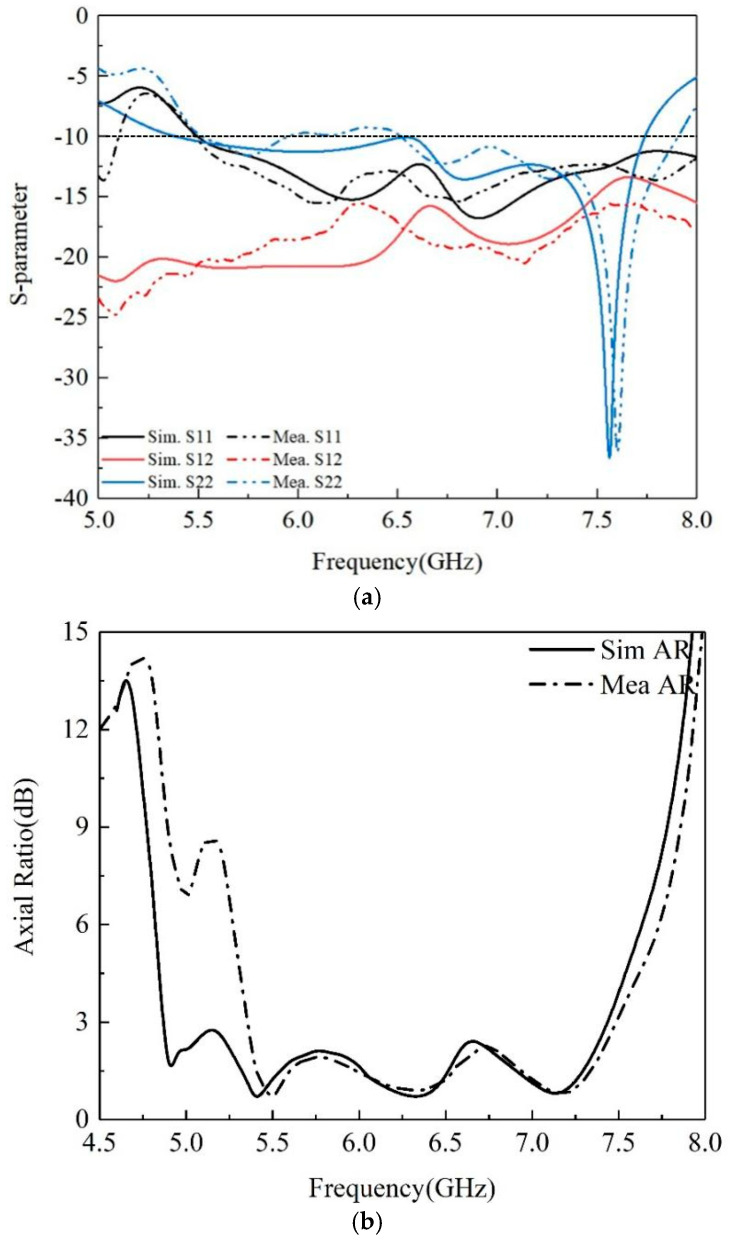
Simulated and measured (**a**) S-parameter between the ports. (**b**) Realized AR of the proposed antenna.

Figure 7a shows the simulated and measured S parameters of the prototype, and the measured and simulated results are in good agreement. The measured impedance bandwidth of −10 dB is 5.5–7.8 GHz. The isolation between the two feed ports is better than 15 dB, and the maximum value can be up to 23 dB around 7.2 GHz. Moreover, the LP antenna resonates at around 7.5 GHz, which is mainly generated by the structure of the monopole. The connection between the folded arm and concentric ring effectively improves the impedance matching of the antenna and makes the antenna reach the resonant state. The simulated and measured AR bandwidth for the antenna are demonstrated in Figure 7b. We can see that the measured 3 dB AR bandwidth is 34.3% and there is similar behavior between the simulation and measurement. Data differences between simulation and measurement are usually caused by a variety of factors, such as antenna manufacturing, the surrounding environment, testing errors, etc.

**Figure 8:**
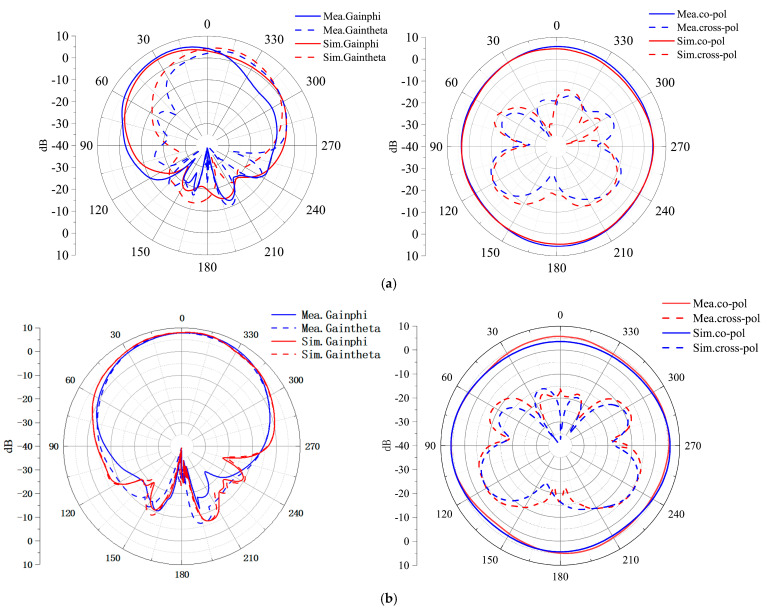
Simulated and measured radiation patterns. (**a**) 5.5 GHz and (**b**) 6.5 GHz.

Figure 8 presents the simulated and measured radiation patterns for two ports at 5.5 GHz and 6.5 GHz. In the band of 5.5–7.8 GHz, the main polarization of CP radiation at port 1 is RHCP, and the radiation for the LP antenna at port 2 can realize omnidirectional radiation. In addition, the cross-polarization levels are generally lower than −10 dB, and the measured results are in good agreement with the simulation.

To draw a better comparison with other existing studies, Table 2 shows the performance of our designed antenna and other dual-polarized antennas, where *λ* is the free space wavelength at the antenna’s lower cutoff frequency. It can be seen that the designed stacked dual-polarized broadband antenna has certain improvements in impedance bandwidth and gain. Moreover, it can also achieve omnidirectional radiation and broadband circular polarization. In summary, the overall performance of the designed antenna has been improved and can be applied in the relay platform or drone detection.

## 4. Conclusions

We design a common-aperture dual-polarized antenna with broadband and low profile by arranging the feed points rationally. Two antennas with different modes of operation are combined on a single PCB, where the CP antenna has an AR bandwidth of 5.3–7.5 GHz and the LP antenna has an impedance bandwidth of 5.5–7.8 GHz. The antenna can support both CP unidirectional radiation and LP omnidirectional radiation within the same frequency band. In addition, the measured results show that the designed antenna produces a peak gain of 8.5 dB at port 1 and 6 dB at port 2. The isolation between the two ports is better than 15 dB. The proposed antenna, with compact size and simple structure, can be applied in space-limited devices for wireless communication applications.

## Figures and Tables

**Table 1:** Optimized numerical parameters of the antenna (units: mm).

Variable	Value	Variable	Value	Variable	Value
*W*	35	*r*0	19.5	*X*4	13.32
*L*	45	*r*1	13.8	*L*1	3.69
*a*	4	*r*2	5.9	*L*2	10.94
*b*	1	*r*3	3.8	*L*3	0.68
*W*1	2.7	*H*	5	*L*4	9.96
*W*2	10.6	*H*1	7	*L*5	11.59
*W*3	3	*H*2	8.5	*L*6	2.2
*X*1	0	*Y*1	4.9	*L*7	21
*x*0	1.1	*y*0	2.2	*X*5	2.9
*X*2	12.6	*X*3	1		

**Table 2:** Comparison of antenna performance between the literature and our design.

Ref.	Size (λ^3^)	BW (%)	Peak Gain (dBi)	AR BW (%)	Polarization	Operation Band (GHz)
[2]	0.616 × 0.616 × 0.095	38.9	6.43	--	L/L	4.45–6.6
		41.4				4.64–7.06
[4]	0.8 × 0.8 × 0.27	6.5	7.2	--	L/L	2.38–2.54
		28.7				3.11–4.15
[11]	0.53 × 0.72 × 0.14	25.6	8	--	L/L	3.3–4.27
		28.5				3.3–4.4
[20]	1.02 × 1.02 × 0.23	48.7	8.15	--	L/L	1.66–2.73
		48.7				1.66–2.73
This work	0.82 × 0.82 × 0.18	34.6	8.5	34.3	C/L	5.5–7.8
		34.6				5.5–7.8

## Data Availability

The original contributions presented in the study are included in the article, further inquiries can be directed to the corresponding author.
